# Clandestinovirus: A Giant Virus With Chromatin Proteins and a Potential to Manipulate the Cell Cycle of Its Host *Vermamoeba vermiformis*

**DOI:** 10.3389/fmicb.2021.715608

**Published:** 2021-08-10

**Authors:** Clara Rolland, Julien Andreani, Dehia Sahmi-Bounsiar, Mart Krupovic, Bernard La Scola, Anthony Levasseur

**Affiliations:** ^1^Aix-Marseille Université (AMU), UMR MEPHI (Microbes, Evolution, Phylogeny and Infections), IRD, APHM, Faculté de Médecine, Marseille, France; ^2^IHU Méditerranée Infection, Marseille, France; ^3^Archaeal Virology Unit, Institut Pasteur, Paris, France; ^4^Institut Universitaire de France, Paris, France

**Keywords:** giant virus, nucleocytoviricota, clandestinovirus, histones, mitochondria, amoeba

## Abstract

For several decades, the vast world of DNA viruses has been expanding constantly. Various discoveries in this field have broadened our knowledge and revealed that DNA viruses encode many functional features, which were once thought to be exclusive to cellular life. Here, we report the isolation of a giant virus named “clandestinovirus,” grown on the amoebal host *Vermamoeba vermiformis*. This virus was discovered in a mixed co-culture associated with another giant virus, Faustovirus ST1. Clandestinovirus possesses a linear dsDNA genome of 581,987 base pairs containing 617 genes. Phylogenetically, clandestinovirus is most closely related to Acanthamoeba castellanii medusavirus and was considered a member of the proposed *Medusaviridae* family. However, clandestinovirus genome is 65% larger than that of medusavirus, emphasizing the considerable genome size variation within this virus family. Functional annotation of the clandestinovirus genes suggests that the virus encodes four core histones. Furthermore, clandestinovirus appears to orchestrate the cell cycle and mitochondrial activities of the infected host by virtue of encoding a panel of protein kinases and phosphatases, and a suite of functionally diverse mitochondrial protein homologs, respectively. Collectively, these observations illuminate a strategy employed by clandestinovirus to optimize the intracellular environment for efficient virus propagation.

## Introduction

Exploration of the giant viruses in amoeba began in 2003 with the discovery of *Acanthamoeba polyphaga mimivirus* (La Scola et al., 2003). Currently, the giant viruses are classified into the phylum *Nucleocytoviricota*, previously designated as Nucleo-cytoplasmic large DNA viruses (NCLDV) (Iyer et al., 2001; Walker et al., 2019; Koonin et al., 2020). Due to their large genome and virion sizes, and unprecedented gene composition, the giant viruses, in many ways, have redefined the whole concept of a “virus.” Indeed, some giant viruses can possess thousands of genes (Colson et al., 2011; Philippe et al., 2013) with, notably, elements of the translation apparatus, such as tRNA, aminoacyl tRNA synthetase (aaRS) or translation factors (Raoult et al., 2004; Schulz et al., 2017; Abrahão et al., 2018). Recent progress in metagenomics and sequence assembly made it possible to detect and reconstruct *in silico* complete genomes of giant viruses from diverse environments (Moniruzzaman et al., 2020a; Schulz et al., 2020b). This contribution enabled the discovery of unexpected metabolic pathways and functions, which were once thought not to be encoded by viruses. Indeed, channelrhodopsins and key enzymatic pathways, such as tricarboxylic cycle, traditionally attributed to cellular life, were discovered in viral genomes, revealing the potential roles played by those giant viruses in their ecosystems (Yutin and Koonin, 2012; Needham et al., 2019a, b). Moreover, giant viruses have been shown to be integrated in the genomes of green algae (Moniruzzaman et al., 2020b) and fungi (Gong et al., 2020). This phenomenon appears to be widespread and highlights the need to further characterize those viruses in order to fully understand their global contributions to the evolution and ecology of eukaryotes.

Some viruses, namely, those belonging to the *Marseilleviridae* family, encode histone doublets in their genomes (Boyer et al., 2009; Thomas et al., 2011). Recently, it has been demonstrated experimentally that histones encoded by two different marseilleviruses localize to the virus factories in the cell cytoplasm and form nucleosomes remarkably similar to those of eukaryotes (Liu Y. et al., 2021; Valencia-Sánchez et al., 2021). Acanthamoeba castellanii medusavirus (medusavirus), a giant virus discovered in a Japanese hot spring, and medusavirus stheno were also shown to possess the four core histones and a linker histone H1 (Yoshikawa et al., 2019; Yoshida et al., 2021). Notably, unlike most other giant viruses, including marseilleviruses, which replicate exclusively in the cytoplasm, replication of the medusavirus genome takes place in the host nucleus (Yoshikawa et al., 2019).

In 2019, we reported the isolation of a potential novel virus, which was mixed with faustovirus ST1 (Rolland et al., 2019). Using single-cell microaspiration applied on amoeba, we succeeded in separating the two viruses (Sahmi-Bounsiar et al., 2019). Here, we describe this new giant virus, named clandestinovirus, discovered in *Vermamoeba vermiformis*. Clandestinovirus shares genes and displays phylogenetic relationship to the Acanthamoeba castellanii medusavirus, suggesting that it is a distant new member of the proposed *Medusaviridae* family. Among the notable features of clandestinovirus, we highlight the presence of genes encoding histones and proteins involved in histone tail modification.

## Materials and Methods

### Sample Collection, Virus Isolation, Production and Purity Control

The co-culture method associated with the single cell micro-aspiration was used to isolate Clandestinovirus in sample from wastewater in Saint-Pierre-de-Mézoargues, France. *Vermamoeba vermiformis* (strain CDC19) was used as cell support. After 48 h in Peptone Yeast extract Glucose medium (PYG) amoebas were washed, harvested at a concentration of 1 × 10^6^ cells/ml and pelleted at 720 × g for 10 min. The cells were re-suspended in the starvation medium at a concentration of 1 × 10^6^ cells/ml (Bou Khalil et al., 2016). Then, the viral sample was inoculated on the cell support at a multiplicity of infection (MOI) of 0.01 and incubated at 30°C until cytopathic effects (CPE) were induced. At this stage, Clandestinovirus was present in our culture but mixed with faustovirus ST1 (Louazani et al., 2017). To separate the two viruses, we used the single cell micro-aspiration method (Sahmi-Bounsiar et al., 2019). Prior to the micro-aspiration, an end-point dilution (10^–1^ to 10^–11^) of the mixed viruses was made in Petri dishes containing 2 ml of amoebas at 1 × 10^6^ cells/ml and 100 μl of the viral mix. At the appearance of the rounding of cells (CPE), the micro-aspiration was performed. Briefly, single infected amoebas from the prepared Petri dishes was picked and cloned into a new Petri dish containing a fresh monolayer of *V*. *vermiformis*, then incubated at 30°C. We monitored the emergence of cytopathic effect characterized by rounding cells daily with an inverted optical microscope and screened the positive culture by standard PCR using specific primers against faustovirus (targeting the *rpb2* gene) and against clandestinovirus (minor capsid protein encoding gene). When we succeeded in separating the two viruses, we proceeded to the viral strain production of clandestinovirus. In brief, 15 flasks of 150 cm^2^ (Corning^®^, NY, United States), infected with the positive culture of clandestinovirus only, were pelleted using the Beckman coulter^®^ Optima^TM^ XPN-80 ultracentrifuge (Beckman Coulter, France) at 50,000 × *g* for 45 min. We finished the purification of the virus using a sucrose gradient of 25% in order to perform whole genome sequencing.

### Replicative Cycle

We use the same protocol as with cedratvirus (Andreani et al., 2016) for the infectious cycle description and the electron microscopy. Briefly, five flasks each containing 30 ml of amoebas at a concentration of 5 × 10^5^ cells/ml were infected with the pure viral suspension at a MOI of 10. After 40 min of incubation, amoebas were washed three times with PAS buffer to eliminate non-internalized viruses. This time point was designated as H0. New flasks were filled with 10 ml of the infected cultures and incubated at 30°C. A flask of non-infected amoebas was used as negative control. At 0, 2, 4, 6, 7, 8, 9, 10, 11, 12 13, 14, 16, 20, and 24 h post infection (hpi), each culture flask corresponding to the specific time point was centrifugated at 720 × *g* for 10 min, and the pellets were fixed for the transmission electron microscopy.

### Genome Sequencing and Genome Assembly

Genomic DNA (gDNA) of clandestinovirus was extracted in two steps: a mechanical treatment was first performed by glass beads acid washed (G4649-500g Sigma) using a FastPrep^TM^-24 5G Grinder (mpBio) at maximum speed (6.5) for 90 s. Then after 30 min lysozyme incubation at 37°C, DNA was extracted on the EZ1 biorobot (Qiagen) with EZ1 DNA tissues kit. The elution volume is 50 μL. gDNA of clandestinovirus was quantified by a Qubit assay with the high sensitivity kit (Life technologies, Carlsbad, CA, United States) to 0.2 ng/μl. Genomic DNA was next sequenced on the MiSeq Technology (Illumina Inc., San Diego, CA, United States) with the paired end strategy and was barcoded in order to be mixed, respectively, with 24 other genomic projects prepared with the Nextera XT DNA sample prep kit (Illumina). To prepare the paired end library, dilution was performed to require 1 ng of each genome as input to prepare the paired end library. The tagmentation step fragmented and tagged the DNA. Then limited cycle PCR amplification (12 cycles) completed the tag adapters and introduced dual-index barcodes. After purification on AMPure XP beads (Beckman Coulter Inc., Fullerton, CA, United States), the libraries were then normalized on specific beads according to the Nextera XT protocol (Illumina). Normalized libraries were pooled into a single library for sequencing on the MiSeq. The pooled single strand library was loaded onto the reagent cartridge and then onto the instrument along with the flow cell. Automated cluster generation and paired end sequencing with dual index reads were performed in a single 39-h run in 2 × 250-bp. Total information of 4.97 Gb was obtained from a 536,000 cluster density per mm^2^ with a cluster passing quality control filters of 94.32%. Within this run, the index representation for clandestinovirus was determined to index 5.78%. The 10,397,314 paired end reads were filtered according to the read qualities.

The genome was assembled using HybridSpades using the default parameters (Antipov et al., 2016).

### Genome Analysis

Gene prediction was computed using GenemarkS (Besemer et al., 2001) and Prodigal v2.6.2 software (Hyatt et al., 2010). The predicted proteins that were smaller than 50 amino acids were deleted. Those between 50 and 99 amino acids were analyzed with Phyre2 software and eliminated of the dataset if they had an abnormal tri-dimensional folding (i.e., proteins with a confidence cut-off under 70% were discarded) (Kelley et al., 2015). A BlastP analysis was performed against the non-redundant (nr) protein database (5 July 2020) using an e-value cut-off of 1E-3. The annotation was performed using a combination of CD-search tool (Marchler-Bauer et al., 2017) and Interpro version 81.0 (Mitchell et al., 2019). In addition, sensitive profile-profile comparisons were performed using HHsearch against the pfam, CDD and COG databases (Steinegger et al., 2019). The core genome analysis was conducted using ProteinOrtho v6 with the parameters of 60% coverage and 20% identity (Lechner et al., 2011). The comparison was performed between clandestinovirus and other giant viruses classified by family. The virus groups included were Acanthamoeba castellanii medusavirus, *Mimiviridae*, klosneuviruses, *Marseilleviridae*, *Pithoviridae*, pandoraviruses, and molliviruses in one group, and *Asfarviridae*, faustoviruses, kaumoebavirus, and pacmanvirus A23 as another group (Supplementary Table 1).

### Phylogenetic Analysis

All phylogenetic analyses were performed using the following procedure. A BlastP with the predicted protein limited to viruses in the NCBI non-redundant (nr) protein database, using an *e*-value cut-off of 1E-3, was computed to retrieve homologous proteins. We kept an average of 65 hits per query protein (ranging between 78 and 57) except for the A32 packaging ATPase and RPB5, where only 49 and 38 homologous proteins were retrieved. For the phylogeny of histones, BlastP was conducted against the non-redundant protein database retaining around 30 hits. Then Muscle program was used to align the sequences (Edgar, 2004). Phylogenetic trees were constructed with the FastTree software using the Jones-Taylor-Thornton (JTT) model for amino acid substitution and the maximum likelihood method with 1,000 bootstrap replications (Price et al., 2010). The trees were visualized using iTOL v6 (Letunic and Bork, 2021).

## Results

### Viral Isolation

The sequencing of the faustovirus ST1 genome revealed the presence of a second genome in the sample. The classical method of giant virus isolation by end-point dilution was used to separate the two viruses in co-culture but without success. A new, recently developed technique, single cell micro-aspiration, was then used allowing the separation of faustovirus ST1 and the second virus, named clandestinovirus (Sahmi-Bounsiar et al., 2019; Supplementary Figure 1). After separation, the new virus was cultivated on *V. vermiformis*, the only known host of Faustovirus, but no lysis was observed. Instead, the infection of *V*. *vermiformis* by clandestinovirus led to rounding of the cells around 8 h post-infection (hpi.) and their detachment after 13 hpi.

### Description of the Replication Cycle

**FIGURE 1:**
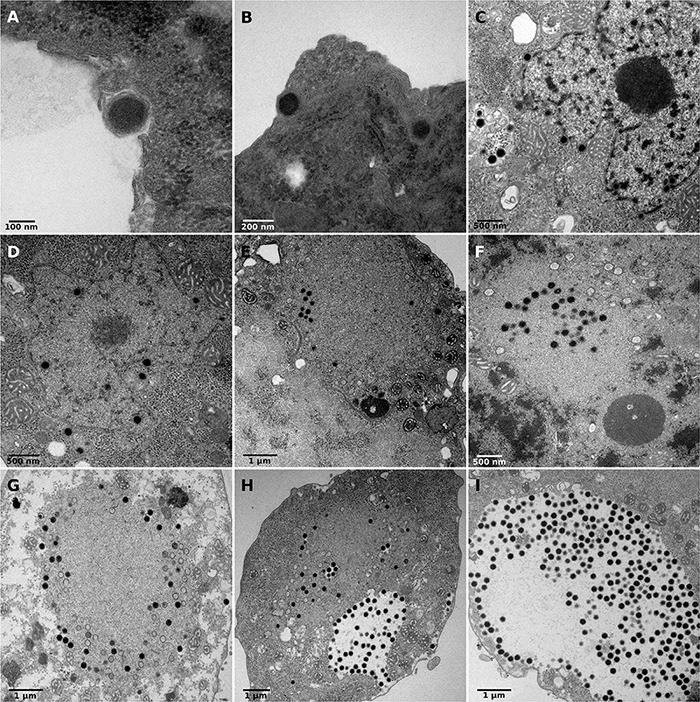
Replicative cycle of clandestinovirus. Scale bars are indicated on each panels. **(A,B)** Entry of the virus into *Vermamoeba vermiformis* by phagocytosis at 4 hpi. **(C,D)** Migration in the cytoplasm to the nucleus and entry into the host nucleus at 7 and 8 hpi. **(E–G)** Shows the replication of clandestinovirus in the nucleus which became a viral factory at 10 hpi **(E)** and 12 hpi **(F,G)**. **(H,I)** Accumulation of mature viral particles at 11 and 12 hpi.

Transmission electron microscopy was used to measure viral particles and describe the replicative cycle of clandestinovirus. The virus has an icosahedral capsid without fibrils. The virion size ranges between 175 and 202 nm with a mean size of 180 nm (SD ± 12 nm; *N* = 71). Briefly, the replicative cycle begin with the entry of the virus into amoeba by phagocytosis (Figures 1A,B). Once inside the cell, between 4 and 7 hpi., the virus migrates through the cytoplasm to the nucleus. At this stage, the clandestinovirus particles cling against the nuclear membrane before the entry of the virus into the nucleus (Figures 1C,D). The replication takes place in the nucleus, which is converted into a viral factory, from 7 to 12 hpi. (Figures 1E–G) as it could observed for Medusavirus in *Acanthamoeba* (Yoshikawa et al., 2019). We clearly see the filing of capsids in the Figure 1G. From 10 hpi., mature virus particles accumulate outside the viral factory, in the cytoplasm of the host *V*. *vermiformis* (Figures 1H,I). These areas of accumulation of mature particles were not observed during the Medusavirus infection where virions were more dispersed in the cytoplasm. The newly assembled virions were released by exocytosis around 16 hpi. In the course of infection, the cells became rounded, lost their adhesion and remained in this state even after 7 days of infection monitored by inverted microscope.

### Genomic Characterization of Clandestinovirus

Clandestinovirus possesses a linear genome of 581,987 base pairs (bp) composed of two scaffolds of 562,042 and 19,445 bp with a G + C content estimated at 43.5 and 46%, respectively (Table 1). A BlastN search of the whole genome predicted 652 ORF but 35 were discarded due to an abnormal tri-dimensional folding (proteins with predictive 3D structure under a confidence cut-off of 70% were removed). Ultimately, 617 genes were retained with a distribution between the two scaffolds of 559 on scaffold 1 and 17 on scaffold 2, corresponding to a global coding proportion of 86.8% of the whole genome (504,963 bp). Two tRNAs were also retrieved, one was a serine tRNA (Ser-tRNA) and the other was a pseudo tRNA with 32% similarity to histidine tRNA (His-tRNA).

**TABLE 1:** Genomic characteristics of clandestinovirus.

**Main genomic characteristics**	
Genome size (bp)	581,987
GC content (%)	43.5
Predicted proteins	617
ORFans (%)	≈ 65.3
Coding density	86.80%
tRNA	2

**FIGURE 2:**
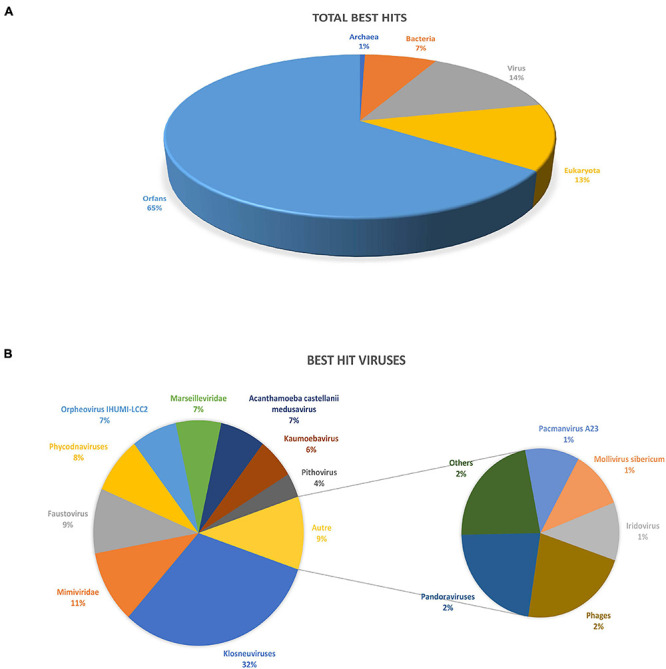
Description of clandestinovirus best hits obtained by blastp against the NCBI non-redundant database. **(A)** Total number of best hits by blast. **(B)** Description of the viruses’ best hits.

BlastP against the nr database yielded 214 proteins with at least one homolog (≈34.7% of all predicted proteins) and 403 unmatched proteins, classified as ORFans (≈65.3% of all predicted genes). Of the 214 proteins, 86 had the best hit with viruses (≈40%), 81 with eukaryotes (≈38%) and 47 with prokaryotes (≈22%; 44 best hits with bacteria and three with archaea) (Figure 2A). For the viral hits, 32% of the best hits were with *Klosneuvirinae* subfamily, 11% with other members of the family *Mimiviridae*, 9% with Faustovirus, and 7% with Medusavirus, Orpheovirus and *Marseilleviridae* (Figure 2B). Regarding the best hits retrieved in Eukaryotes, the majority (30%) were with Metazoans, 22% with Fungi, and 12% with Viridiplantae. For the prokaryotes, most hits were with Bacteroidetes (≈23%), followed by Gammaproteobacteria (≈7%) and Firmicutes and Alphaproteobacteria (≈5% each).

Seven genes were found to carry introns. Five genes coding for DNA-directed RNA polymerase subunit 1 (RPB1), DNA-directed RNA polymerase subunit 2 (RPB2), ribonucleoside-diphosphate reductase large subunit, ankyrin repeat protein and DNA processing protein A (DprA) contained a single intron each. In contrast, the gene encoding a family B DNA polymerase elongation subunit contained two introns (Supplementary Table 2).

The ORF annotation indicated that 5.1% of the clandestinovirus genes correspond to the NCLDV core genes described by Koonin and Yutin (2010; Table 2), including four of the five inferred ancestral NCLDV proteins, namely, major capsid protein, the family B DNA polymerase, the A32-like DNA-packaging ATPase and the viral late transcription factor 3 (VLTF3). In addition to the typical major capsid protein with the double jelly-roll fold, genes for the minor capsid proteins were found. They are homologous to the Paramecium bursaria chlorella virus 1 (PBCV-1) penton protein with the single jelly-roll fold found at the five-fold vertices of the icosahedral capsid as well as the PBCV-1 proteins P8 and P9, both involved in bridging and stabilizing the capsomers. This strongly suggests that the assembly principle of the clandestinovirus capsid is similar to that of phycodnaviruses and, likely, most other NCLDVs.

**TABLE 2:** Nucleo-cytoplasmic large DNA viruses (NCLDV) core genes in the clandestinovirus genome.

**Clandestinovirus NCLDV core genes**		

***ORF n*°**	***NCLDV gene annotation***	***Associated functions***
203, 509	DNA or RNA helicase of superfamily II	DNA replication, recombination, and repair
315	D5-like helicase primase	DNA replication, recombination, and repair
211,212,213	DNA polymerase elongation subunit family B	DNA replication, recombination, and repair
299	ATP-dependent DNA ligase	DNA replication, recombination, and repair
582	FLAP-like endonuclease XPG	DNA replication, recombination, and repair
406	DNA topoisomerase II	DNA replication, recombination, and repair
49, 50	Ribonucleotide diphosphate reductase large subunit	Nucleotide metabolism
20	Ribonucleotide reductase small subunit	Nucleotide metabolism
524	Thymidylate synthase	Nucleotide metabolism
73, 389, 435	Dual specificity phosphatases; Ser/Thr, and Tyr proteins phosphatases	Other metabolic functions
269	Ubiquitin-conjugating enzyme 2	Other metabolic functions
445, 446	DNA-directed RNA polymerase subunit alpha	Transcription and RNA processing
25, 26	DNA-directed RNA polymerase subunit beta	Transcription and RNA processing
75	Divergent DNA-directed RNA polymerase subunit 5	Transcription and RNA processing
437	mRNA capping enzyme large subunit	Transcription and RNA processing
219, 573	Nudix hydrolase	Transcription and RNA processing
84	RNA ligase	Transcription and RNA processing
365	Transcription factor S-II (TFIIS)	Transcription and RNA processing
537	Poxvirus late transcription factor VLTF3 like	Transcription and RNA processing
364	Disulfide oxidoreductase; Erv1/Alr family	Virion structure and morphogenesis
154, 366	NCLDV major capsid protein	Virion structure and morphogenesis
13	A32-like packaging ATPase	Virion structure and morphogenesis

The clandestinovirus genome encodes several proteins involved in metabolic pathways. Nicotinamide/nicotinate mononucleotide adenylyl transferase (NMNAT) and NADAR (NAD^+^ and ADP-ribose) participate in NAD^+^ biosynthesis and NAD^+^-utilization pathway, whereas cystathionine gamma-synthase and dihydrofolate reductase are involved in the synthesis of cystathionine and purines, respectively. Interestingly, we also identified a homolog of RNase T2, which has been recently described in Tupanvirus soda lake and discovered in Fadolivirus (Rolland et al., 2020; Schulz et al., 2020a).

We found that the virus encodes 11 serine/threonine kinases (Supplementary Table 3), including homologs of the cell cycle-related kinases, and three protein phosphatases, including mitogen-activated protein phosphatases. Remarkably, clandestinovirus encodes two homologs of Cyclin A2 (Cheng et al., 2006; Zhang et al., 2019), a factor that regulates cell cycle progression by interacting with two different cyclin-dependent kinases (CDK): CDK2 during S phase and CDK1 during the transition from G2 to M phase (Pagano et al., 1992). In addition, clandestinovirus encodes a homolog of Cdc123, an ATP-grasp fold protein involved in cell cycle control by promoting the transition from G1 to S phase (Panvert et al., 2015). Thus, clandestinovirus potentially orchestrate the cell cycle through controlling the phosphorylation state of key cellular factors as well as by direct interaction with the cellular or viral factors.

Furthermore, five LAGLIDADG endonuclease and two HNH family intron-encoded homing endonuclease were identified, which might have a potential impact on introns. Similar to phycodnaviruses, clandestinovirus encodes one DNA methyltransferase and eight restriction endonucleases of the HNH, PD-(D/E)XK and ββα-Me families which might be involved in the degradation of cellular DNA (Agarkova et al., 2006; Coy et al., 2020). In addition, the virus encodes a homolog of the mimivirus R354-like nuclease, a part of the MIMIVIRE system, with the closest homologs in phycodnaviruses (Levasseur et al., 2016). Moreover, several proteins with functions linked to the nucleus and DNA replication/transcription were identified. These include four BTB/POZ proteins (transcriptional regulators), several transcription factors as well as histone-like proteins.

Interestingly, the virus also encodes 10 proteins functioning in mitochondria (Supplementary Table 4), including three copies of the mitochondrial chaperone BCS1, which translocate substrates across the mitochondrial inner membrane without previous unfolding (Kater et al., 2020); mitochondrial deoxyguanosine kinase; Dynamin 1-like protein (DNM1-L), a mechanochemical GTPase that induces membrane fission in mitochondria (Ford et al., 2011); mitochondrial sulfhydryl oxidase Erv1p; mitochondrial aspartate/glutamate carrier protein Aralar/Citrin; and two copies of the mitochondrial/bacterial CCA-adding enzyme.

### Phylogenetic Relationship to Other Known Giant Viruses

A core and pan-genome analyses were carried out to determine the relationship of clandestinovirus to the previously characterized giant viruses (Supplementary Table 5). We considered a gene to belong to a cluster of orthologous genes (COG) if at least one reciprocal best hit was obtained between clandestinovirus and one virus included in the family tested. The largest number of conserved genes is shared with the klosneuviruses, with a total of 58 COGs, followed by the *Mimiviridae* (42 COG). Somewhat surprisingly, only 16 COGs are shared between clandestinovirus and medusavirus, despite the phylogenetic proximity of their core genes.

**FIGURE 3:**
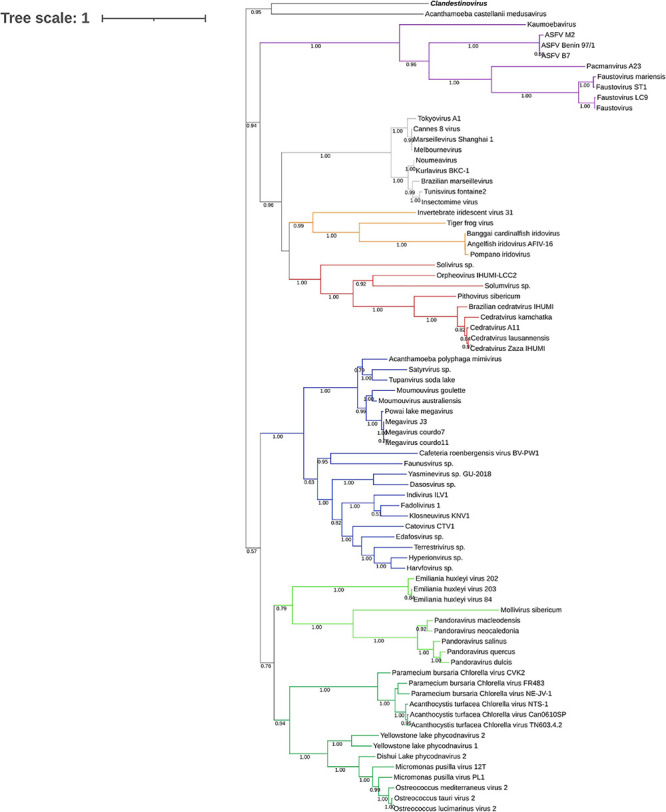
Phylogenetic tree of the DNA polymerase B sequences. The analysis was performed using the Maximum Likelihood method (ML) with a JTT substitution matrix in 1,000 replicates. Branch values lower than a bootstrap value of 0.5 were deleted. Colors were assigned for different group of viruses: blue for Mimiviruses and extended *Mimiviridae*; green for Pandoraviruses, Mollivirus sibericum, Emiliania huxleyi viruses (light green) and *Phycodnaviridae* (dark green); purple for groups of *Asfarviridae*, Faustoviruses, Pacmanvirus, and Kaumoabevirus; gray for Marseilleviridae; red for Orpheovirus, Solumvirus, Solivirus, Cedratviruses, and Pithovirus sibericum; and orange for *Iridoviridae*. Clandestinovirus is highlighted in bold and italic.

To determine the placement of clandestinovirus among other giant viruses, phylogenetic analysis was performed on nine conserved proteins. First, we chose three proteins considered to be ancestral to all NCLDVs and commonly used to determine the phylogenetic relationships among giant viruses (family B DNA polymerase, major capsid protein and VLTF-3). We added six more proteins commonly encoded in giant virus genomes (RPB1, RPB2, RPB5, and A32-like genome packaging ATPase as well as large and small subunits of the ribonucleoside-diphosphate reductase) (Koonin and Yutin, 2019). Phylogenetic analysis has shown that in most gene trees clandestinovirus forms a sister group to medusavirus (Figure 3 and Supplementary Figures 2–9). Notably, however, the genome of clandestinovirus is 65% larger than that of medusavirus (581,987 versus 381,277 bp), indicating that the two viruses are only distantly related, consistent with a limited number of shared COGs (see above). Distant relationship is also supported by the observation that genes with introns are different between the two viruses, except for those encoding the family B DNA polymerase elongation subunit and the large subunit of the ribonucleoside-diphosphate reductase. Clandestinovirus was even more distantly related to Emiliania huxleyi viruses, Molliviruses, and Pandoraviruses, being placed at the root of branches including these viruses in five trees out of nine.

### Deciphering Viral Histones and Associated Proteins in Clandestinovirus

Clandestinovirus carries homologs of the genes coding for four core histones (H3, H4, and H2B/H2A) and an additional gene for the linker histone H1/H5. The comparison with giant viruses encoding histones (Marseillevirus, Lausannevirus, and Medusavirus) showed that both Clandestinovirus and Medusavirus encode the four histones and a linker H1/H5, whereas Marseillevirus and Lausannevirus only encode three histones with a histone doublet H2B/H2A, a histone 2A-domain-containing protein and the histone H3 (Table 3).

**TABLE 3:** Description of proteins associated with histones in Clandestinovirus, Marseillevirus, Lausannevirus, and Acanthamoeba castellanii medusavirus.

**Clandestinovirus**		**Marseillevirus marseillevirus**		**Lausannevirus**		**Acanthamoeba castellanii medusavirus**	

**Histones**							

**Protein ID**	**Annotation**	**Protein ID**	**Annotation**	**Protein ID**	**Annotation**	**Protein ID**	**Annotation**
CV_ORF456	Histone H2B/H2A	YP_003407138.1	Histone H2B/H2A fusion protein	YP_004347348.1	Histone H2B/H2A fusion protein	BBI30201.1	Histone H2B
CV_ORF417	Histone H3	YP_003407137.1	Histone H3	YP_004347349.1	Histone H3-like protein	BBI30395.1	Histone H3
CV_ORF467	Histone H4					BBI30394.1	Histone H4
CV_ORF99	Linker histone H1/H5					BBI30246.1	Linker histone H1
		YP_003406909.1	Histone 2A-domain-containing protein	YP_004347019.1	Histone 2A-domain-containing protein	BBI30458.1	Histone H2A

**FIGURE 4:**
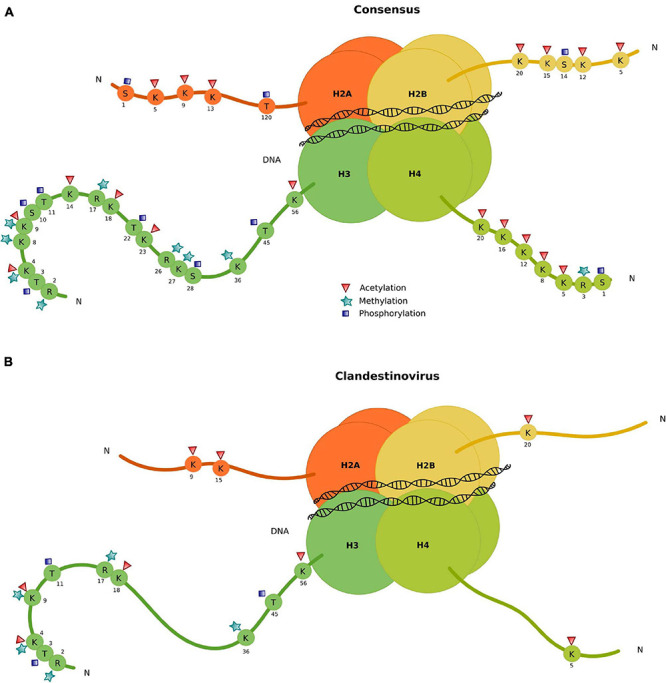
Conservation of specific modification sites in N-terminal tails of clandestinovirus histones. **(A)** Consensus. **(B)** Histone conserved modification sites in clandestinovirus.

To understand how clandestinovirus might benefit from encoding its own histones, we investigated the conservation of specific modification sites in N-terminal histone tails by aligning their sequences with sequences of histones from eukaryotes (Figure 4 and Supplementary Table 6). The alignment of sequences for histone H2B and H4 showed two conserved sites of tail modification: the lysine 20 in position 3 of the H2B domain and lysine 5 in H4. For the histone domain H2A, lysines in positions 5 and 11 of the H2A domain from clandestinovirus coincide with lysines 9 and 15 of the consensus H2A sequence. Those two positions are the acetylation sites. The most conserved sites of modification are in the histone H3 with five sites of methylation on arginine 2 and lysines 4, 9, and 36. There are also five sites of acetylation (lysines 4, 9, 14, and 18) and one site of phosphorylation (threonine 11).

**FIGURE 5:**
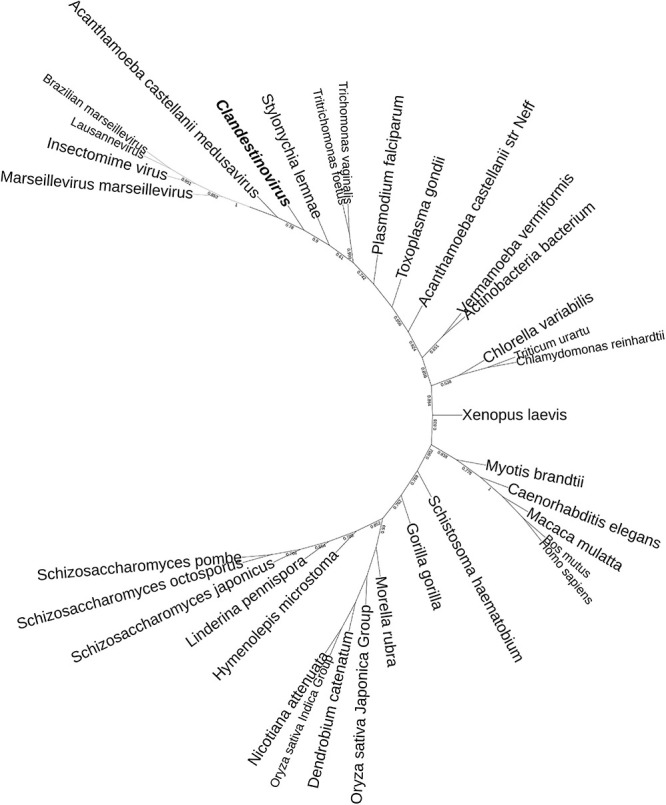
Phylogenetic tree of the histone H3 of clandestinovirus. The analysis was performed using the ML method with a Jones-Taylor-Thornton (JTT) substitution matrix and 1,000 replicates. Branches with bootstrap support values below 0.50 were collapsed. Clandestinovirus is highlighted in bold and italic.

The phylogeny of histones indicates a branching of clandestinovirus between a clade including medusavirus and *Marseilleviridae* on one side and eukaryotic sequences on the other side (Figure 5). The phylogenetic distance between the histones of clandestinovirus and the host *V*. *vermiformis* suggested an acquisition from a source other than an amoebal host.

## Discussion

In this work, we described a novel giant virus isolated on *V*. *vermiformis* and named clandestinovirus ST1. It is a virus that was discovery in co-culture with another giant virus, faustovirus ST1 (Louazani et al., 2017). Surprisingly, clandestinovirus is not lytic and is released without causing a fast lysis of its host. It showed a viral cycle similar to that described for Acanthamoeba castellanii medusavirus, a giant virus recently discovered in Japan (Yoshikawa et al., 2019). Both viruses have an unusual replication cycle when compared to other giant viruses, with the entry into the nucleus of their amoebal hosts. However, unlike medusavirus, clandestinovirus replication, including virion assembly, takes place in the viral factories formed directly inside the nucleus.

Seven introns have been identified in the genome of clandestinovirus, as many as in medusavirus. In addition, seven homing endonucleases have been detected, five of the LAGLIDADG family and two of the HNH family. These enzymes bind and cleave DNA in specific patterns, providing the location for the insertion of intron-creating sequences if they are in coding regions (Stoddard, 2005), which might allow for the insertion of the introns.

Many viruses are known to manipulate the cell cycle to ensure the most favorable intracellular conditions for virus replication (Bagga and Bouchard, 2014; Fan et al., 2018; Liu J. et al., 2021). Interestingly, clandestinovirus encodes two cyclins with homologs in giant viruses and herpesviruses. Viral cyclins have been studied in the case of herpesviruses and were shown to bind to one or more cellular cyclin-dependent kinases and phosphorylate an increased number of substrates compared with their cellular counterparts, thereby aiding virus replication (Hardwick, 2000; Ojala et al., 2000; Verschuren et al., 2004). Notably, clandestinovirus encodes multiple protein kinases and dephosphatases, which could further promote virus replication through orchestration of the cell cycle. Furthermore, the virus encodes several proteins functioning in mitochondria, among which mitochondrial chaperone BCS1 has been also detected in the medusaviruses but in single copy (Yoshikawa et al., 2019; Yoshida et al., 2021). The presence of mitochondria surrounding viral factories might indicate that these proteins help to stimulate some mitochondrial activities. Collectively, these observations suggest that clandestinovirus takes control of the cell cycle and optimizes the mitochondrial functions to boost its replication. Recent findings with Acanthamoeba castellanii medusavirus support this hypothesis (Zhang et al., 2021). Indeed, the authors have shown an alteration of the transcription of nuclear genes and a maintenance of the genes associated with mitochondria.

The number of COGs shared with the other giant virus families is inconsistent with the phylogeny of the core genes. Indeed, clandestinovirus shares the highest number of genes with klosneuviruses and tupanvirus, while in phylogenies, it forms a sister group to the medusavirus, despite disparity in genome sizes between the two viruses. One explanation is that the pan-genome of klosneusviruses is much larger than that of other virus groups and that overrepresentation of klosneusviruses in the database introduces a bias.

Furthermore, the annotation of clandestinovirus revealed the presence in its genome of numerous genes encoding proteins with functions associated or linked to the nucleus. Indeed, we found transcriptional factors, transcriptional regulators and proteins necessary for the viral DNA replication and transcription. Among them, we also retrieved homologs of the four histones, one of which is a histone doublet H2B/H2A, and the linker H1/H5. The presence of these proteins suggests that the virus might form nucleosomes, a complex associating DNA and histones. This complex is subject to post-translational modifications which either activate or repress the transcription of DNA necessary for viral replication (Kouzarides, 2007). In particular, these modifications include methylation and acetylation at particular residues located within histone tails and are well described in eukaryotic organisms. The analysis of these modification sites in clandestinovirus point to a conservation of these marks mostly on histone H3. They notably encompass acetylations, methylations or phosphorylations on well identified amino acids (Huang et al., 2014). In general, histone acetylation enables the activation of transcription, especially on lysine 56 of histone H3 (H3K56) (Tessarz and Kouzarides, 2014). A high level of acetylation combined with trimethylation of H3K4, H3K36, and H3K79 corresponds to actively transcribed euchromatin. In contrast, methylation sites on lysines H3K9, H3K27, and H4K20 are linked to the inhibition of transcription (Kouzarides, 2007). In clandestinovirus, only three activation sites (H3K4, H3K36, and H3K56) and one repressive site (H3K9) are conserved. Phylogenetic analysis of the viral histone places clandestinovirus at the base of the viral clade including medusavirus and marseilleviruses.

Moreover, histones are not the only elements associated with viral DNA replication found in clandestinovirus. Indeed, a potential restriction-modification system (R-M system) including the DNA *N*-6-adenine methyltransferase (Dam) and type-II restriction endonucleases were identified (eight HNH restriction endonucleases of different families). This system is linked to DNA methylation and is widely found in giant viruses (Jeudy et al., 2020). Some authors suggest that it could be a protection system against the host or other microorganisms (viruses or bacteria). In chloroviruses, the R-M system is involved in the degradation of cellular DNA to allow viruses to use the nucleotide pools created (Agarkova et al., 2006). In the case of clandestinovirus, the R-M system might be either a protection system that make it possible to coexist with faustovirus ST1 and a weapon to allow it to have access to more nucleotides for its replication.

Clandestinovirus is a new giant virus discovered in *V. vermiformis*. Its replicative cycle takes place in the host nucleus, and its genomic characteristics and phylogeny suggest that it is a distant relative of Acanthamoeba castellanii medusavirus. We suggest that clandestinovirus represents a separate genus within the family *Medusaviridae* proposed by Yoshikawa et al. (2019). Clandestinovirus opens new perspectives for this family, broadening the host range to a new amoeba species. Further investigations on the diversity of the *Medusaviridae* family should expand our knowledge on the co-evolution between large DNA viruses and eukaryotes.

## Data Availability Statement

The data presented in the study are deposited in the NCBI database repository under accession numbers MZ420154 and MZ420155.

## Author Contributions

AL and BL designed and supervised the study. CR, JA, DS-B, MK, and AL performed sample collection, virus isolation, experiments, and analyses. CR, JA, MK, and AL wrote the manuscript. All authors read and approved the final version of the manuscript.

## Conflict of Interest

The authors declare that the research was conducted in the absence of any commercial or financial relationships that could be construed as a potential conflict of interest.

## Publisher’s Note

All claims expressed in this article are solely those of the authors and do not necessarily represent those of their affiliated organizations, or those of the publisher, the editors and the reviewers. Any product that may be evaluated in this article, or claim that may be made by its manufacturer, is not guaranteed or endorsed by the publisher.
