# Supplementary material for: Clandestinovirus: A Giant Virus With Chromatin Proteins and a Potential to Manipulate the Cell Cycle of Its Host Vermamoeba vermiformis
Source: Front Microbiol. 2021 Aug 10;12:715608. doi: 10.3389/fmicb.2021.715608 (PMC8383183; doi:10.3389/fmicb.2021.715608)
Supplement: Supplementary file 3 [file Table_3.docx]

**Table S3**. Clandestinovirus proteins potentially involved in cell cycle control.

| **Protein** | **Manually verified annotation** | **HHsearch hit** | **Probability** |
| --- | --- | --- | --- |
| CV_ORF22 | Serine/Threonine kinase | cd14070 STKc_HUNK; Catalytic domain of the Serine/Threonine Kinase, Hormonally up-regulated Neu-associated kinase (also called MAK-V). | 99.97 |
| CV_ORF42 | Serine/Threonine kinase | cd08215 STKc_Nek; Catalytic domain of the Serine/Threonine Kinase, Never In Mitosis gene A (NIMA)-related kinase. | 100 |
| CV_ORF44 | Serine/Threonine kinase | cd08215 STKc_Nek; Catalytic domain of the Serine/Threonine Kinase, Never In Mitosis gene A (NIMA)-related kinase. | 99.97 |
| CV_ORF73 | Dual specificity protein phosphatase | 2Y96_A dual specificity phosphatase dupd1 {Homo sapiens} | 99.85 |
| CV_ORF180 | Cyclin A2 involved in cell division checkpoint control | 6Q6G_S Cell division cycle protein 20; spindle assembly checkpoint, anaphase-promoting complex {Homo sapiens} | 99.95 |
| CV_ORF232 | Serine/Threonine kinase | cd08215 STKc_Nek; Catalytic domain of the Serine/Threonine Kinase, Never In Mitosis gene A (NIMA)-related kinase. | 100 |
| CV_ORF309 | Serine/Threonine kinase | cd08215 STKc_Nek; Catalytic domain of the Serine/Threonine Kinase, Never In Mitosis gene A (NIMA)-related kinase. | 100 |
| CV_ORF318 | Serine/Threonine kinase | cd08215 STKc_Nek; Catalytic domain of the Serine/Threonine Kinase, Never In Mitosis gene A (NIMA)-related kinase. | 100 |
| CV_ORF328 | Serine/Threonine kinase | cd07830 STKc_MAK_like; Catalytic domain of Male germ cell-Associated Kinase-like Serine/Threonine Kinases. | 100 |
| CV_ORF354 | Serine/Threonine kinase | cd06640 STKc_MST4; Catalytic domain of the Serine/Threonine Kinase, Mammalian Ste20-like protein kinase 4. | 99.98 |
| CV_ORF356 | Serine/Threonine kinase | cd08215 STKc_Nek; Catalytic domain of the Serine/Threonine Kinase, Never In Mitosis gene A (NIMA)-related kinase. | 100 |
| CV_ORF389 | Serine/Threonine phosphatase | KOG0697 Protein phosphatase 1B (formerly 2C) | 99.97 |
| CV_ORF435 | Dual specificity protein phosphatase | 2G6Z_B Dual specificity protein phosphatase 5; alpha/beta, HYDROLASE {Homo sapiens} | 99.89 |
| CV_ORF548 | Cyclin A2 involved in cell division checkpoint control | 6Q6G_S Cell division cycle protein 20; spindle assembly checkpoint, anaphase-promoting complex {Homo sapiens} | 100 |
| CV_ORF577 | Cdc123 from Schizosaccharomyces pombe; ATP-grasp fold; cell cycle proliferation | 4ZGN_A Full-length Cdc123 from Schizosaccharomyces pombe; ATP-grasp fold, cell cycle, eIF2 {Schizosaccharomyces pombe} | 100 |
| CV_ORF579 | Serine/Threonine kinase | cd07830 STKc_MAK_like; Catalytic domain of Male germ cell-Associated Kinase-like Serine/Threonine Kinases. | 100 |
| CV_ORF580 | Serine/Threonine kinase | cd14169 STKc_CaMKI_beta; Catalytic domain of the Serine/Threonine kinase, Calcium/calmodulin-dependent protein kinase Type I beta. | 99.98 |
