# Supplementary material for: Clandestinovirus: A Giant Virus With Chromatin Proteins and a Potential to Manipulate the Cell Cycle of Its Host Vermamoeba vermiformis
Source: Front Microbiol. 2021 Aug 10;12:715608. doi: 10.3389/fmicb.2021.715608 (PMC8383183; doi:10.3389/fmicb.2021.715608)
Supplement: Supplementary file 4 [file Table_4.DOCX]

**Table S4.** Clandestinovirus proteins potentially involved in mitochondrial activities.

| **Protein** | **Manually verified annotation** | **Hhsearch hit** | **Probability** |
| --- | --- | --- | --- |
| CV_ORF93 | Mitochondrial chaperone BCS1 | 6UKO_G Mitochondrial chaperone BCS1 {Mus musculus} | 100 |
| CV_ORF221 | Deoxyguanosine kinase, mitochondrial precursor | 2OCP_G Deoxyguanosine kinase, mitochondrial precursor {Homo sapiens} SCOP: c.37.1.1 | 99.77 |
| CV_ORF273 | Dynamin 1-like protein (DNM1-L), mechanochemical GTPase that induces membrane fission in mitochondria | 3ZVR_A dynamin-1 (E.C.3.6.5.5); DRP1, DRP, mitochondrial endocytosis {Rattus norvegicus} | 100 |
| CV_ORF303 | Mitochondrial chaperone BCS1 | 6SH4_G Mitochondrial chaperone BCS1 {Saccharomyces cerevisiae} | 99.98 |
| CV_ORF364 | Mitochondrial sulfhydryl oxidase Erv1p | 1JR8_A Erv2 PROTEIN, mitochondrial; FAD, sulfhydryl oxidase {Saccharomyces cerevisiae} SCOP: a.24.15.1 | 99.92 |
| CV_ORF506 | Mitochondrial aspartate/glutamate carrier protein Aralar/Citrin | KOG0751 Mitochondrial aspartate/glutamate carrier protein Aralar/Citrin (contains EF-hand Ca2+-binding domains) | 100 |
| CV_ORF539 | Mitochondrial chaperone BCS1 | 6SH4_G Mitochondrial chaperone BCS1 {Saccharomyces cerevisiae} | 100 |
| CV_ORF559 | Mitochondrial/bacterial CCA-adding enzyme [ATP (CTP): tRNA nucleotidyltransferase, hCCAase] | cd05398 NT_ClassII-CCAase; Nucleotidyltransferase (NT) domain of Class II CCA-adding enzymes. CCA-adding enzymes add the sequence [cytidine(C)-cytidine-adenosine (A)], one nucleotide at a time, onto the 3' end of tRNA, in a template-independent reaction | 97.19 |
| CV_ORF570 | Mitochondrial/bacterial CCA-adding enzyme [ATP (CTP): tRNA nucleotidyltransferase, hCCAase] | cd05408 NT_hCCAase_like; NT_hCCAase_like. NT_hCCAase_like: Human mitochondrial CCA-adding enzyme [ATP (CTP): tRNA nucleotidyltransferase, hCCAase]-like, class II. | 98.29 |
