# Supplementary material for: Clandestinovirus: A Giant Virus With Chromatin Proteins and a Potential to Manipulate the Cell Cycle of Its Host Vermamoeba vermiformis
Source: Front Microbiol. 2021 Aug 10;12:715608. doi: 10.3389/fmicb.2021.715608 (PMC8383183; doi:10.3389/fmicb.2021.715608)
Supplement: Supplementary file 6 [file Table_6.DOCX]

**Table S6.** Localisation and types of modifications in tail histones present in clandestinovirus.

| **Histones** | **Amino acid** | **Position** | **Type of modification** |
| --- | --- | --- | --- |
| **H2A** | Lysine (K) | 9 | Acetylation |
|  | Lysine (K) | 13 | Acetylation |
| **H2B** | Lysine (K) | 20 | Acetylation |
| **H3** | Arginine (R) | 2 | Methylation |
|  | Threonine (T) | 3 | Phosphorylation |
|  | Lysine (K) | 4 | Acetylation/Methylation |
|  | Lysine (K) | 9 | Acetylation/Methylation |
|  | Threonine (T) | 11 | Phosphorylation |
|  | Arginine (R) | 17 | Methylation |
|  | Lysine (K) | 18 | Acetylation |
|  | Lysine (K) | 36 | Acetylation |
|  | Threonine (T) | 45 | Phosphorylation |
|  | Lysine (K) | 56 | Acetylation |
| **H4** | Lysine (K) | 5 | Acetylation |
