# Supplementary material for: Clandestinovirus: A Giant Virus With Chromatin Proteins and a Potential to Manipulate the Cell Cycle of Its Host Vermamoeba vermiformis
Source: Front Microbiol. 2021 Aug 10;12:715608. doi: 10.3389/fmicb.2021.715608 (PMC8383183; doi:10.3389/fmicb.2021.715608)
Supplement: Supplementary file 8 [file Table_8.DOCX]

Supplementary Material

**Figure S1.** Negative staining of clandestinovirus.

**Figure S2.** Phylogenetic tree based on MCP of nucleo-cytoplasmic large DNA viruses. Branch values lower than a bootstrap value of 0.5 were deleted. Colors were assigned for different group of viruses: blue for Mimiviruses and extended *Mimiviridae*; green for Mollivirus sibericum (light green) and *Phycodnaviridae* (dark green); purple for groups of *Asfarviridae* and Faustoviruses; grey for Marseilleviridae; red for Orpheovirus, Cedratviruses and Pithovirus sibericum. Clandestinovirus is highlighted in bold and italic.

**Figure S3.** Phylogenetic tree based on VLTF3 protein of nucleo-cytoplasmic large DNA viruses. Branch values lower than a bootstrap value of 0.5 were deleted. Colors were assigned for different group of viruses: blue for Mimiviruses; green for Pandoraviruses, Mollivirus sibericum (light green) and Phycodnaviridae (dark green); purple for groups of Asfarviridae, Faustoviruses and Pacmanvirus; grey for Marseilleviridae; red for Orpheovirus, Solumvirus, Solivirus, Cedratviruses and Pithovirus sibericum. Clandestinovirus is highlighted in bold and italic.

**Figure S4.** Phylogenetic tree based on RPB1 protein of nucleo-cytoplasmic large DNA viruses. Branch values lower than a bootstrap value of 0.5 were deleted. Colors were assigned for different group of viruses: blue for Mimiviruses and extended *Mimiviridae*; green for Pandoraviruses, Molliviruses, Emiliania huxleyi viruses (light green) and *Phycodnaviridae* (dark green); purple for groups of *Asfarviridae*, Faustoviruses, Pacmanvirus and Kaumoabevirus; grey for Marseilleviridae; red for Orpheovirus, Cedratviruses and Pithovirus sibericum and orange for Asco-Iridoviridae. Clandestinovirus is highlighted in bold and italic.

**Figure S5.** Phylogenetic tree based on RPB2 protein of nucleo-cytoplasmic large DNA viruses. Branch values lower than a bootstrap value of 0.5 were deleted. Colors were assigned for different group of viruses: blue for Mimiviruses and extended *Mimiviridae*; green for Pandoraviruses, Molliviruses, Emiliania huxleyi viruses (light green) and *Phycodnaviridae* (dark green); purple for groups of *Asfarviridae*, Faustoviruses, Pacmanvirus and Kaumoabevirus; grey for Marseilleviridae; red for Orpheovirus, Cedratviruses and Pithovirus sibericum and orange for Iridoviridae. Clandestinovirus is highlighted in bold and italic.

**Figure S6.** Phylogenetic tree based on RPB5 protein of nucleo-cytoplasmic large DNA viruses. Branch values lower than a bootstrap value of 0.5 were deleted. Colors were assigned for different group of viruses: blue for Mimiviruses; green for Pandoraviruses (light green) and *Phycodnaviridae* (dark green); purple for groups Faustoviruses and Pacmanvirus; grey for *Marseilleviridae*; red for Cedratvirus and Pithoviruses. Clandestinovirus is highlighted in bold and italic.

**Figure S7.** Phylogenetic tree based on A32-like genome packaging ATPase of nucleo-cytoplasmic large DNA viruses. Branch values lower than a bootstrap value of 0.5 were deleted. Colors were assigned for different group of viruses: blue for Mimiviruses and extended *Mimiviridae*; green for Pandoraviruses, Molliviruses, Emiliania huxleyi viruses (light green) and *Phycodnaviridae* (dark green); purple for groups of Pacmanvirus and Kaumoabevirus and grey for Marseilleviridae. Clandestinovirus is highlighted in bold and italic.

**Figure S8.** Phylogenetic tree based on Ribonucleoside-diphosphate reductase large subunit of nucleo-cytoplasmic large DNA viruses. Branch values lower than a bootstrap value of 0.5 were deleted. Colors were assigned for different group of viruses: blue for Mimiviruses; green for Pandoraviruses, Emiliania huxleyi viruses (light green) and *Phycodnaviridae* (dark green); purple for groups of Faustoviruses and Pacmanvirus; grey for Marseilleviridae; red for Orpheovirus, Cedratviruses and Pithovirus sibericum. Clandestinovirus is highlighted in bold and italic.

**Figure S9.** Phylogenetic tree based on Ribonucleoside-diphosphate reductase small subunit of nucleo-cytoplasmic large DNA viruses. Branch values lower than a bootstrap value of 0.5 were deleted. Colors were assigned for different group of viruses: blue for Mimiviruses and extended *Mimiviridae*; green for Pandoraviruses (light green) and *Phycodnaviridae* (dark green); purple for groups of *Asfarviridae* and grey for Marseilleviridae. Clandestinovirus is highlighted in bold and italic.
