# Supplementary figures and images for: Clandestinovirus: A Giant Virus With Chromatin Proteins and a Potential to Manipulate the Cell Cycle of Its Host Vermamoeba vermiformis
Source: Front Microbiol. 2021 Aug 10;12:715608. doi: 10.3389/fmicb.2021.715608 (PMC8383183; doi:10.3389/fmicb.2021.715608)

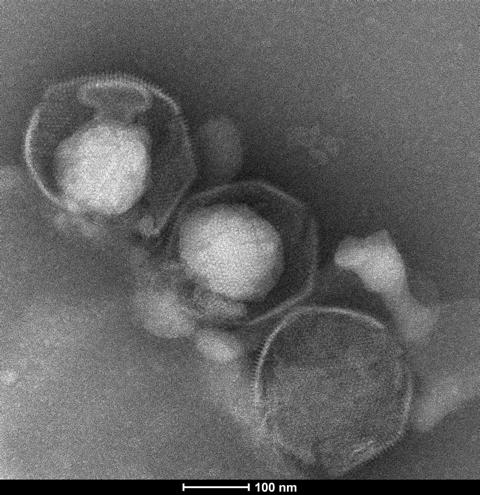

Supplement: Supplementary file 9 [file Image_1.JPEG]

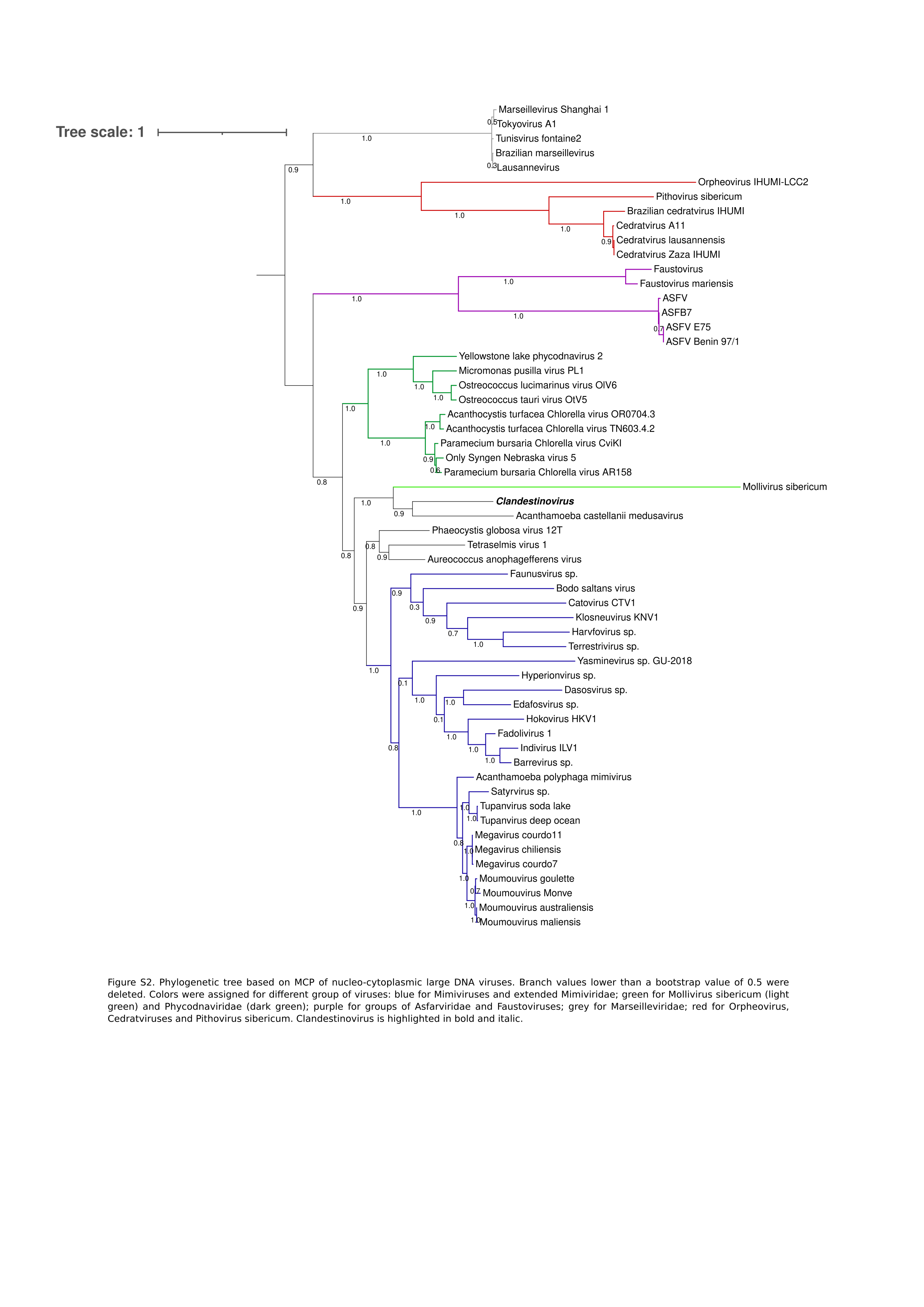

Supplement: Supplementary file 10 [file Image_2.tiff]

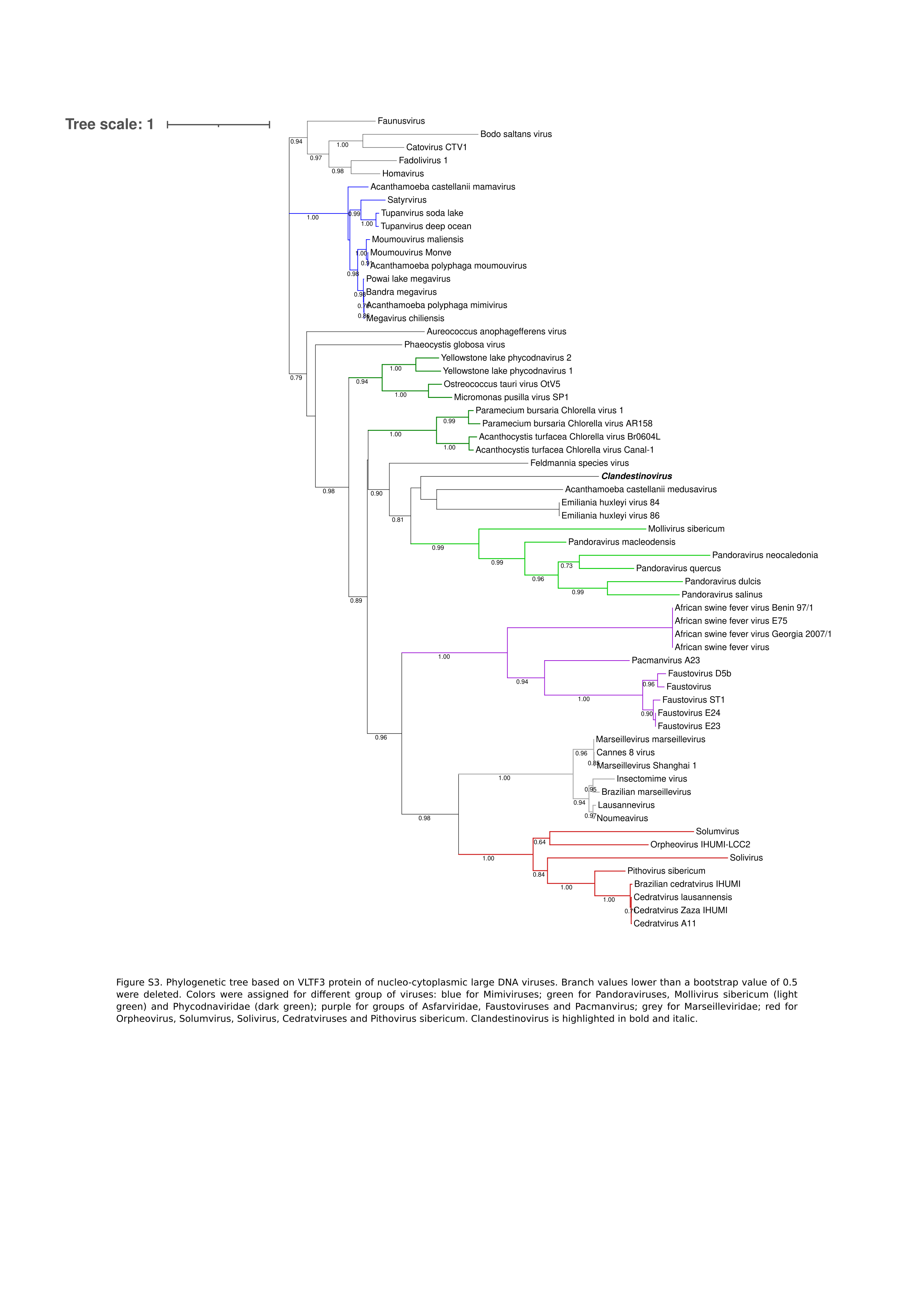

Supplement: Supplementary file 11 [file Image_3.tiff]

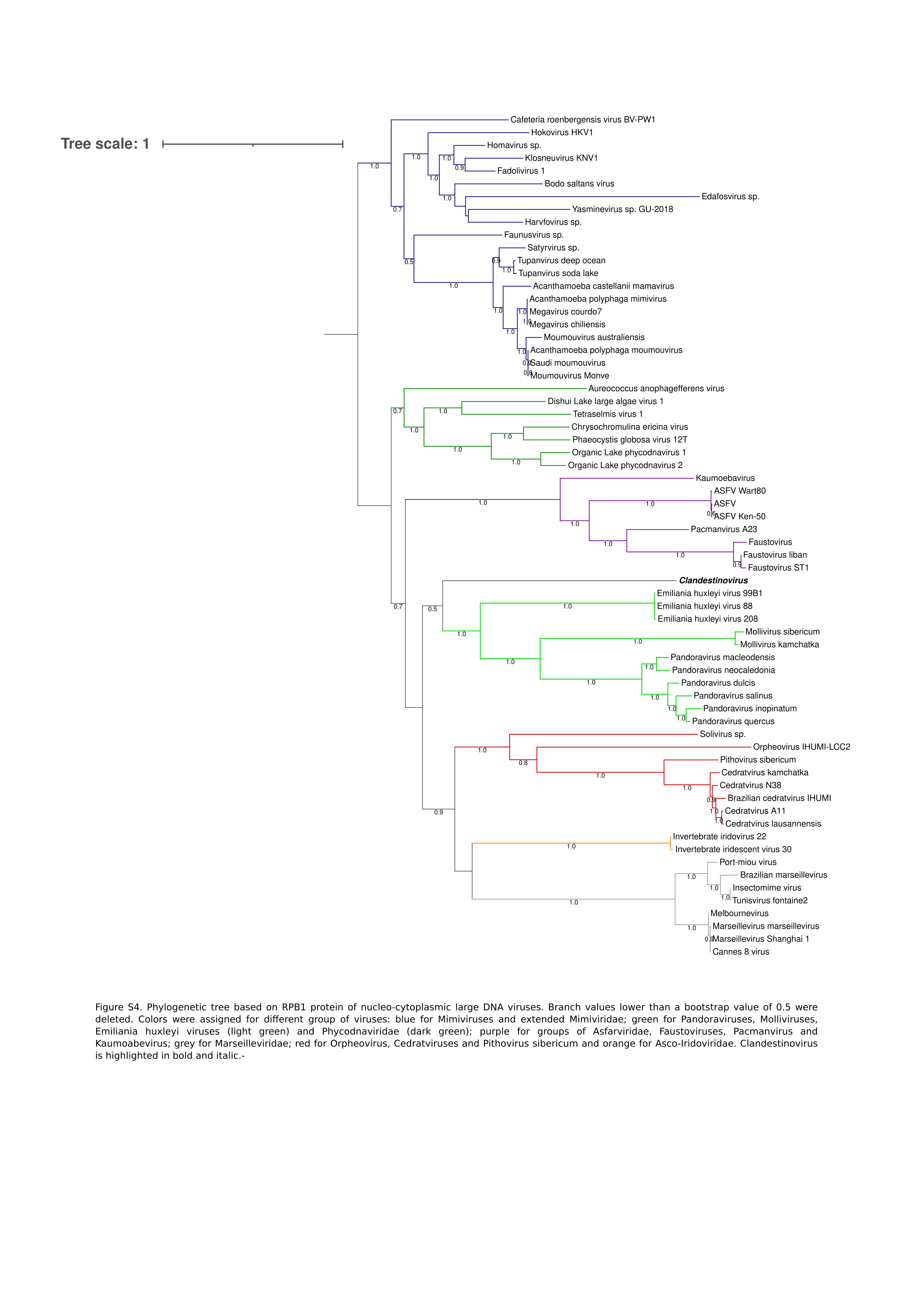

Supplement: Supplementary file 12 [file Image_4.tiff]

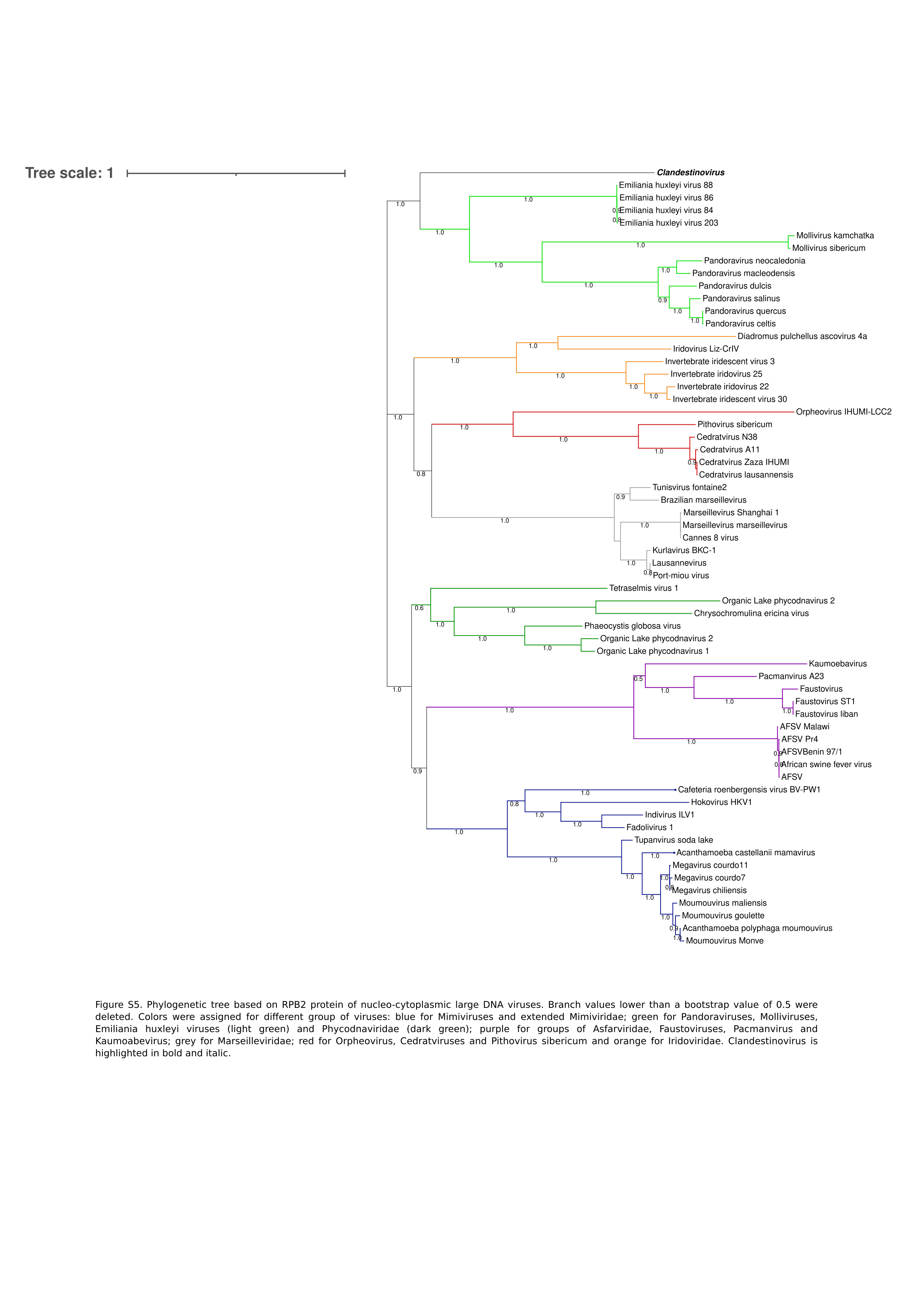

Supplement: Supplementary file 13 [file Image_5.tiff]

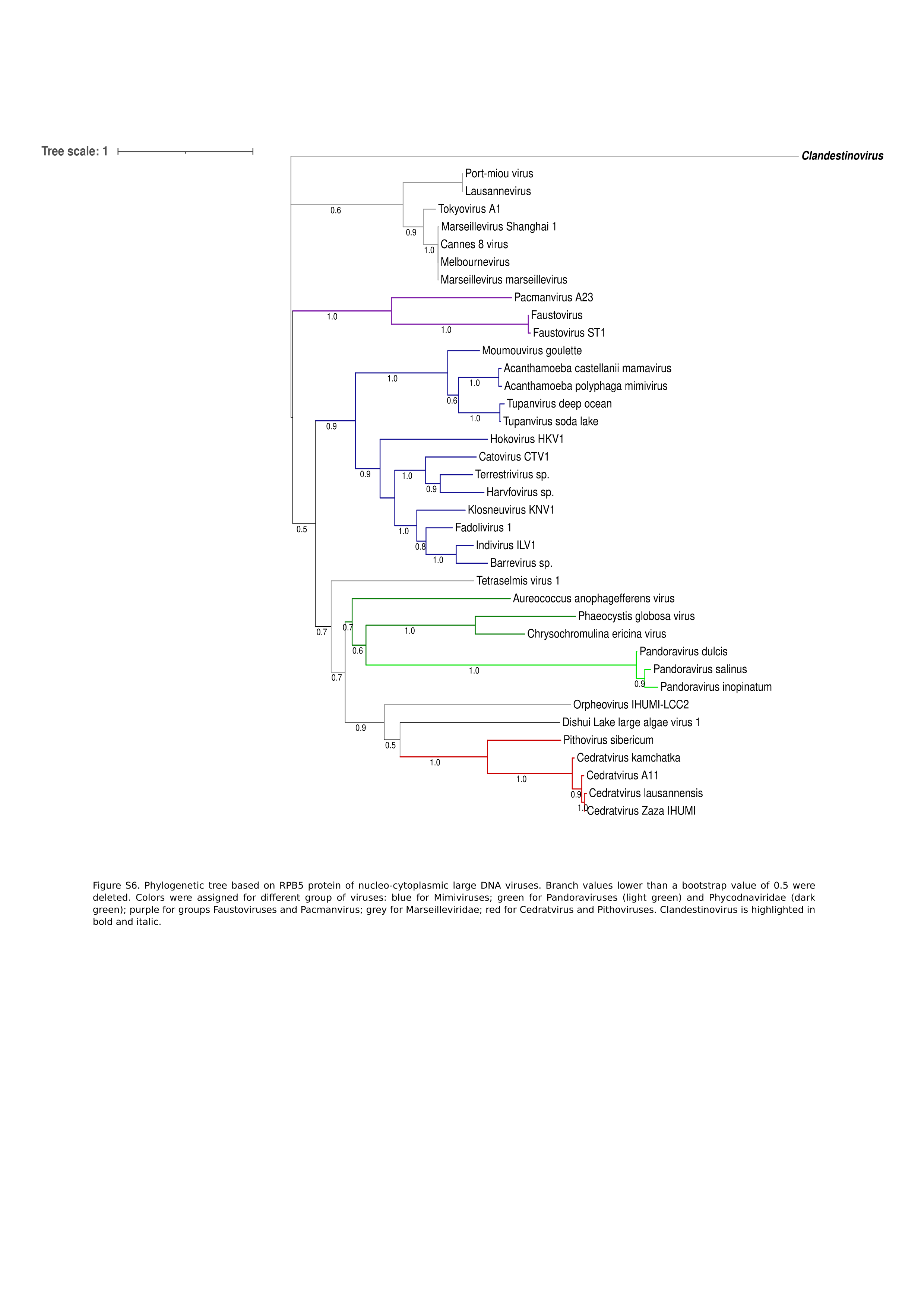

Supplement: Supplementary file 14 [file Image_6.tiff]

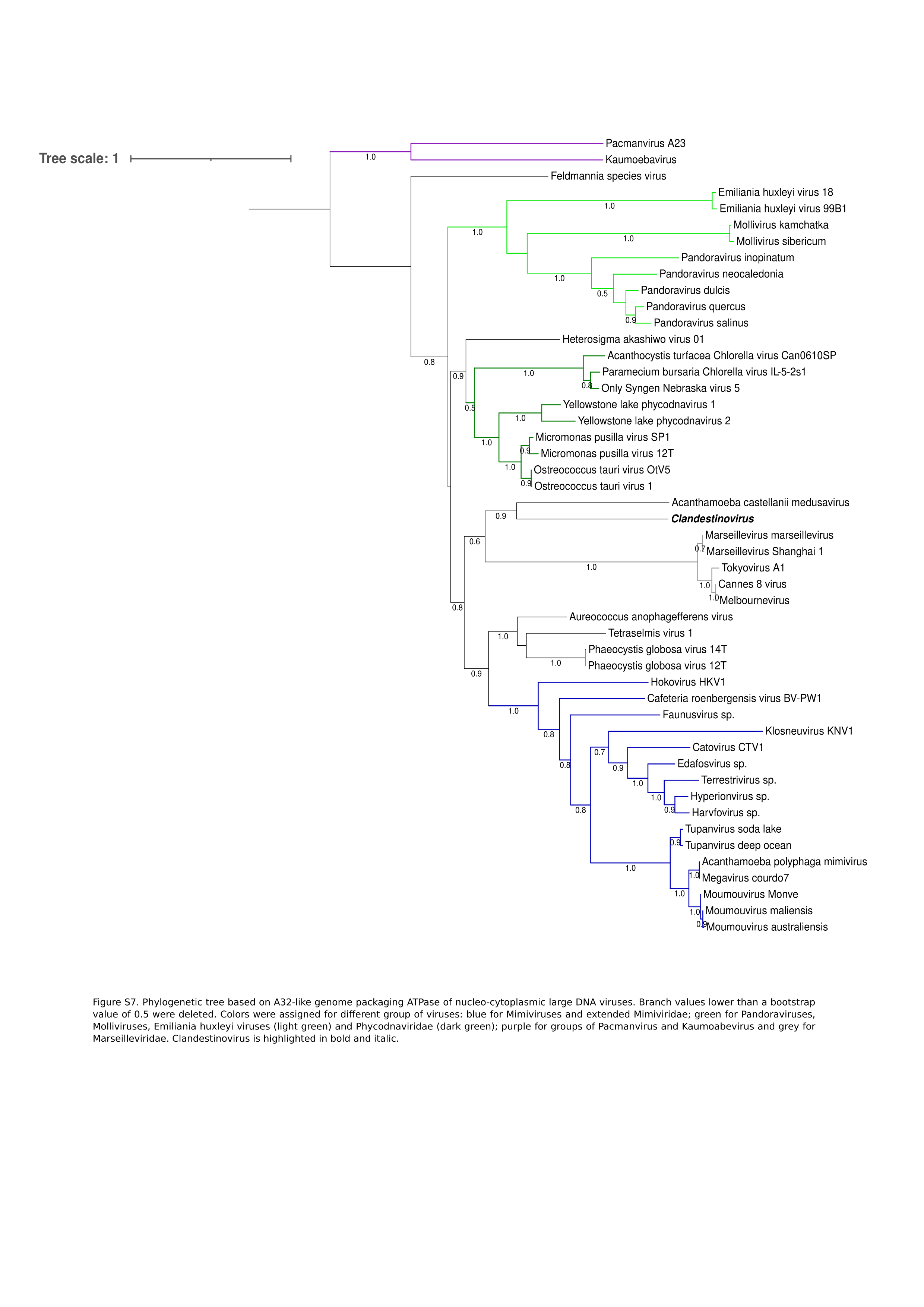

Supplement: Supplementary file 15 [file Image_7.tiff]

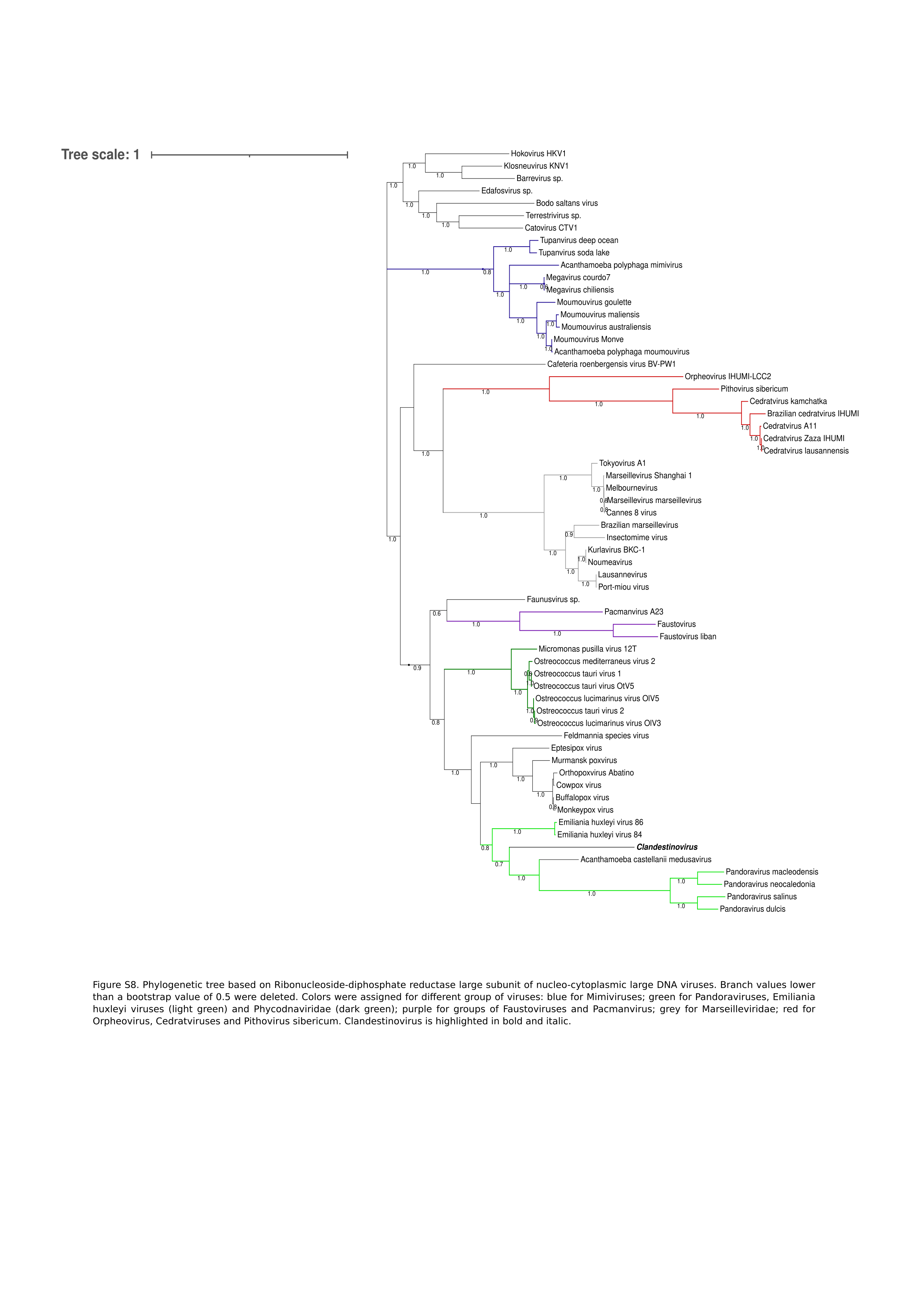

Supplement: Supplementary file 16 [file Image_8.tiff]

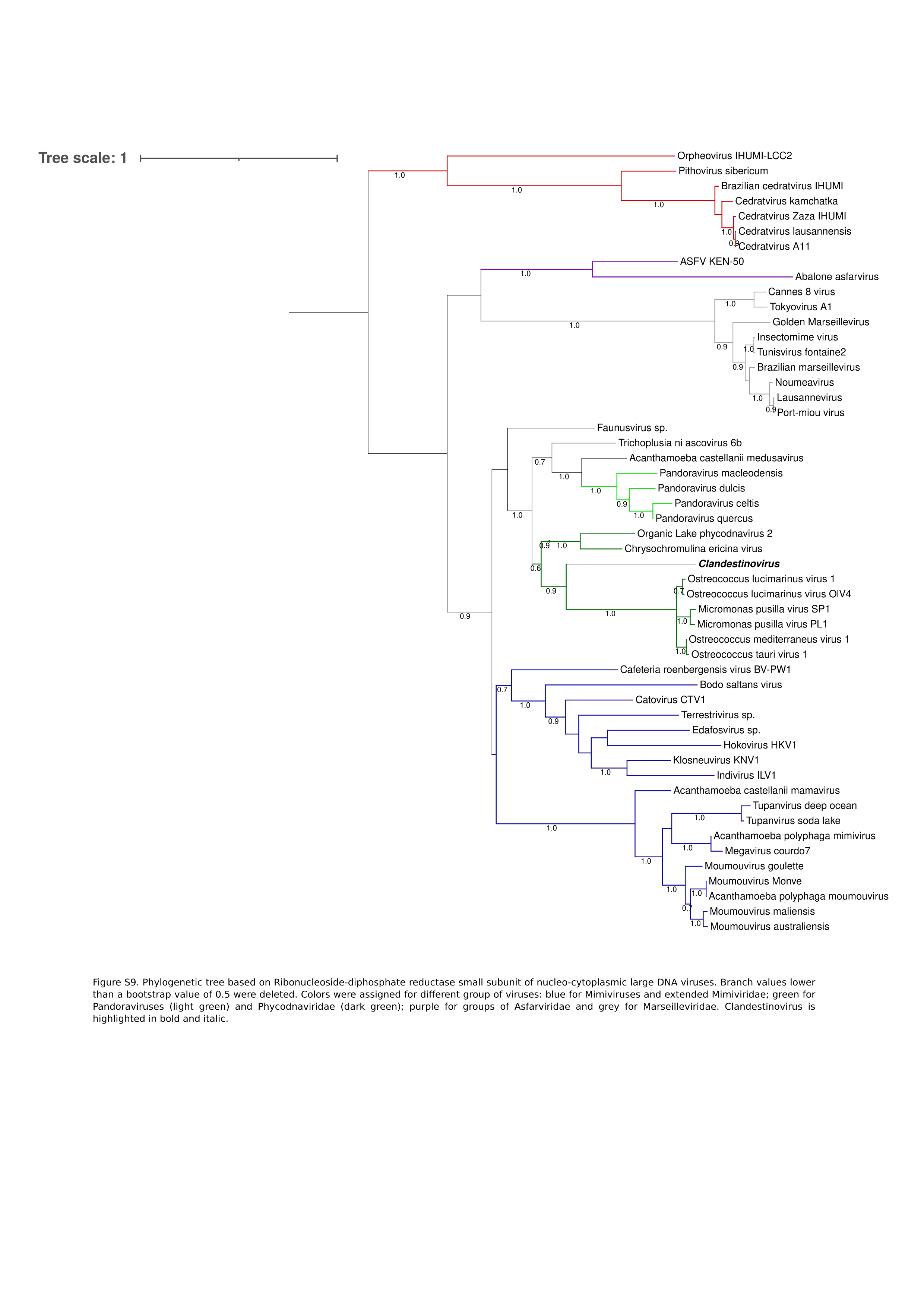

Supplement: Supplementary file 17 [file Image_9.tiff]
